# 3% diquafosol sodium eye drops in Chinese patients with dry eye: a phase IV study

**DOI:** 10.3389/fmed.2023.1089613

**Published:** 2023-05-25

**Authors:** Wenying Wang, Xiaonan Sun, Jiangyue Zhao, Jilong Hao, Shaozhen Zhao, Xiaoming Yan, Ye Shen, Xiuming Jin, Yan Cheng, Linnong Wang, Jianjiang Xu, Peiquan Zhao, Hai Liu, Siming Zeng, Xu Wang, Weili Dong, Jinsong Xue, Wei Chen, Ping Guo, Li Li, Lijun Zhang, Dachuan Liu, Baihua Chen, Zhouqiao Lin, Yanjiang Fu, Lingyi Liang, Yanling Dong, Weizhong Yang, Yingping Deng, Guigang Li, Zhiqiang Pan

**Affiliations:** ^1^Beijing Tongren Eye Center, Beijing Institute of Ophthalmology, Beijing Tongren Hospital, Capital Medical University and Beijing Ophthalmology and Visual Sciences Key Laboratory, Beijing, China; ^2^Department of Ophthalmology, The 4th People's Hospital of Shenyang, Shenyang, China; ^3^Department of Ophthalmology, The Forth Affiliated Hospital of Medical School, Shenyang, China; ^4^Department of Ophthalmology, First Hospital of Jilin University, Jilin, China; ^5^Department of Refractive and Corneal Diseases, Eye Center of Tianjin Medical University, Tianjin, China; ^6^Department of Ophthalmology, Peking University First Hospital, Beijing, China; ^7^Department of Ophthalmology, The First Affiliated Hospital of Medical School of Zhejiang University, Hangzhou, China; ^8^Eye Center, The Second Affiliated Hospital of Medical School of Zhejiang University, Hangzhou, China; ^9^Department of Ophthalmology, Xi'an No. 1 Hospital, Xi’an, China; ^10^Department of Ophthalmology, Nanjing First Hospital, Nanjing, China; ^11^Department of Ophthalmology, Eye and Ent Hospital of Fudan University, Shanghai, China; ^12^Department of Ophthalmology, Xinhua Hospital Affiliated to Shanghai Jiaotong University School of Medicine, Shanghai, China; ^13^Department of Ophthalmology, Second People's Hospital of Yunnan, Kunming, China; ^14^Department of Ophthalmology, The People's Hospital of Guangxi Zhuang Autonomous Region, Nanning City, China; ^15^Department of Ocular Surface Diseases, Jinan Second People's Hospital, Jinan, China; ^16^Department of Ophthalmology, Affiliated Hospital of Chengde Medical University, Chengde, China; ^17^Department of Corneal Diseases, The Affiliated Hospital of Nanjing Medical University, Nanjing, China; ^18^Department of Corneal Diseases, Eye Hospital, WMU, Zhejiang EYE Hospital, Hangzhou, China; ^19^Department of Keratopathy and Ocular Surface Diseases, Shenzhen Eye Hospital, Shenzhen, China; ^20^Department of Cataract Division, Joint Shantou International Eye Center (JSIEC), Shantou University and The Chinese University of Hong Kong, Shantou, China; ^21^Department of Ophthalmology, The Third People's Hospital of Dalian, Dalian, China; ^22^Department of Ophthalmology, Xuanwu Hospital of Capital Medical University, Beijing, China; ^23^Department of Ophthalmology, The Second Hospital of Central South University, Changsha, China; ^24^Department of Ophthalmology, The People's Hospital of Leqing, Wenzhou, China; ^25^Department of Ophthalmology, Daqing Ophthalmologic Hospital, Daqing, China; ^26^Department of Corneal Diseases, Zhongshan Ophthalmic Center (ZOC) of Sun YAT-Sen University, Guangzhou, China; ^27^Department of Corneal Diseases, Qingdao Eye Hospital, Qingdao, China; ^28^Department of Ophthalmology, Guangzhou First People's Hospital, Guangzhou, China; ^29^Department of Ophthalmology, West China Hospital, Sichuan University, Chengdu, China; ^30^Department of Ophthalmology, Tongji Hospital Affiliated to Tongji Medical College, Huazhong University of Science and Technology, Wuhan, China

**Keywords:** dry eye, diquafosol sodium eye drops, efficacy, safety, flourescein staining

## Abstract

**Introduction:**

The efficacy and safety of 3% diquafosol sodium eye drops in Chinese patients with dry eye in the real-world setting remains unclear.

**Methods:**

3099 patients with dry eye symptoms were screened according to Asia Dry Eye Society latest recommendation. Among them, 3000 patients were enrolled for a phase IV study. We followed up with multiple clinical characteristics including corneal fluorescein staining, tear break up time, Schirmer’s tests, visual acuity, intraocular pressure, and others. The follow ups were performed at baseline, 2 weeks and 4 weeks after treatment.

**Results:**

Based on the results of corneal fluorescein staining and tear break up time, all age and gender subgroups exhibited obvious alleviation of the symptoms among the patients with dry eye, and the data in elderly group showed the most significant alleviation. All the adverse drug reactions (ADRs, 6.17%) were recorded, among which 6% local ocular ADRs were included. Meanwhile, mild ADRs (91.8%) accounted for the most. Most of the ADRs (89.75%) got a quick and full recovery, with an average time at 15.6 days. 1.37% of patients dropped out of the study due to ADRs.

**Discussion:**

The use of 3% diquafosol sodium eye drop is effective and safe in the treatment of dry eye, with a low incidence of ADRs showing mild symptoms. This trial was registered at Chinese Clinical Trial Registry ID: ChiCTR1900021999 (Registration Date: 19/03/2019).

## Introduction

Dry eye is a multifactorial disease which is characterized by unstable tear film causing a variety of symptoms and/or visual impairment, and it is potentially accompanied by ocular surface damage (1). Although the classification of dry eye could be diversified, the Asia Dry Eye Society (ADES) has proposed a simplified classification (i.e., increased evaporation, aqueous-deficient, and decreased wettability), which makes a practical diagnosis feasible using the simple fluorescein staining patterns of tear film (2). Despite the diversified classification, the main manifestation of dry eye disorder is eye discomfort or blurred vision, and superficial ocular injuries found in objective examinations. With the increase of environmental pollution, the use of makeup, contact lenses and video screen terminals, the dry eye prevalence has increased sharply, and in turn raised economic and psychological burdens rooted in the increased prevalence (3).

Ocular surface desiccation can cause apoptosis in corneal epithelial cells and decrease mucin secretion, leading to corneal or conjunctival damage, which affects the barrier function of the ocular surface epithelium and aggravates dryness (4). If not treated in time, such damage may lead to a dysfunction of the cell connection between the corneal basement membrane and recurring epithelial exfoliation or filamentary keratitis. The pathogenesis might progress into the corneal stroma and become corneal ulcers (3). Therefore, the goal of dry eye treatment is not a temporary relief of symptoms but a sufficient replenishment of tears, a reconstruction of the tear film stability and a recovery of punctate epithelial damage (5).

The tears of healthy individuals contain aqueous, mucus and lipid, as well as proteins, electrolytes and vitamins (6). Dry eye is characterized by a lack of some aqueous- mucous component or increased evaporation of tears in the early stage; with disease progression, there may be a lack of all components. There are at least three types of commercialized artificial tears: viscous lubricating agents that replenish certain tear components (7, 8); drugs that promotes tear secretion, i.e., diquafosol sodium; and drugs that is mainly for anti-inflammation and promoting tear secretion, i.e., cyclosporine. Lubricating artificial tears are used as exogenous supplements, mainly composed of tear aqueous or lipid layer replenisher. Artificial tears can temporarily relieve dry eye symptoms but are not sufficient to repair the punctate stains caused by corneal epithelial damage commonly found in patients with moderate to severe symptoms. Moreover, lubricating artificial tears can only be applied intermittently and as a result, it could only alleviate dry eye symptoms temporarily and not as good as natural tears (5). Therefore, diquafosol sodium eye drops (Santen, firstly approved in Japan in 2010, approved in China in 2017, and approved in 11 countries by November 2021), which can promote endogenous tear components secretion and restore a line of defense for the epithelial cells against infection and injury (9, 10) has emerged. By promoting aqueous, mucin and lipid secretion through P2Y_2_ receptor, diquafosol sodium eye drops can balance the functions of conjunctival goblet cells, the corneal epithelium, meibomian gland sebaceous, and ductal cells (11). Diquafosol sodium can also act on meibomian glands to increase lipid secretion, thereby fully supplementing the three-layer components of the tear film and leads to a full recovery of the tear film stability and rehydration (12).

3% diquafosol sodium eye drops were generally well tolerated, previously reported ADRs associated with diquafosol sodium were eye irritation or foreign body sensation (13). Most ADRs disappeared after a few days, and they are usually mild or moderate. For long-term ADRs, it was reported that eye discharge, eye irritation and eye pain were the major manifestation (14).

We reported a multi-center phase III clinical trial of 3% diquafosol sodium eye drops included 573 dry eye cases in China and Singapore (13), where the benefit of diquafosol sodium eye drops were shown in patients ≥21 years and <70 years. However, patients of different ages, genders or races may respond differently to this type of artificial eye fluid, the phase III trial results beg the question of how 3% diquafosol sodium eye drops could benefit real world patients. In order to comprehensively evaluate the therapeutic effect of 3% diquafosol sodium eye drops on dry eye cases and possible ADRs in more diversified populations, this study expanded the cohort to include patients with mild to moderate dry eye from different regions in multiple provinces of China. With corneal fluorescein staining score as the primary efficacy endpoint according to ADES most updated recommendation (2), combined with other efficacy endpoints of dry eye, this study aimed to evaluate the efficacy and safety of 3% diquafosol sodium eye drops in Chinese patients with mild to moderate dry eye of different ages, genders, and medical histories.

## Methods

### Patients

This was a phase IV open label single arm study. To ensure the maximal heterogeneity of patient enrolled, a total of 30 clinical centers that met the clinical trial’s criteria participated. Participating centers are listed in the Supplemental information (Supplementary Table S1). This study was approved by the Ethics Committee of Beijing Tongren Hospital, Capital Medical University (approval number: TREC2018-50). All patients provided signed informed consent, and the study followed the Declaration of Helsinki.

The inclusion criterion were in accordance with the previous report (13) as follow: at least 18 years of age; male or female; patients with at least one of the subjective symptoms, including dryness, foreign body sensation, burning sensation, fatigue, discomfort, or vision fluctuation; BUT≤5 s or Schirmer I test (without surface anesthesia) results ≤10 mm/5 min; positive corneal fluorescein staining (CFS) (NEI score). The main exclusion criteria were: legal blindness in either eye; a history of allergy to any component of the study drugs or tests (such as diquafosol sodium or fluorescein); use of concomitant ocular or systemic non-steroidal anti-inflammatory drugs, hormones, immunosuppressants, recent use of eye drops (within 1 week), recent physical therapy or eye surgery(within 2 weeks) that would impact the dry eye symptom; recent use of contact lenses (within 2 weeks); punctal occlusion treatments such as lacrimal point embolization or lacrimal point occlusion; independent eye disorder that require additional treatments; pregnancy planning, pregnant or breastfeeding women. We made sure all female patients of childbearing age had a negative urine pregnancy test before enrollment. If both eyes of the patient were eligible for the trial, only the eye with higher corneal fluorescein staining score at baseline was included in the final analysis (to balance the statistical procedure), in case of identical staining of both eyes, the right eye was chosen (Chinese Clinical Trial Registry ID: ChiCTR1900021999, Registration Date: 19/03/2019).

#### ADR assessment

The severity of ADR is a qualitative assessment of the scope or degree of adverse drug reactions, which is divided into three levels of mild, moderate, and severe based on the following criteria:

Mild: The patient has symptoms and signs, but can tolerate them completely.
Moderate: The symptoms and signs cause discomfort to the patient, affecting their normal activities.
Severe: The symptoms and signs prevent the patient from working or carrying out daily activities.

### Therapy regimen and follow up

3% (5 ml:150 mg) diquafosol sodium eye drops [Diquas^®^, provided by Santen Pharmaceutical (China) Co., Ltd] were prescribed for each patient, and they were advised to apply one drop at a time, six times a day, for 1 month.

Follow up examinations were carried out at baseline, 2- and 4-weeks of treatment.

(1) Visual acuity examination. The 5 m standard visual acuity chart was used to measure visual acuity. The results were recorded as decimals and converted into international standard logarithmic visual acuity for statistical analysis. For example, a VA of 0.1 was LogMAR (20/200 in US).
(2) Slit lamp microscopy. A slit lamp microscope was used to observe the conjunctiva, cornea, anterior chamber, and lens, recording potential abnormalities.
(3) BUT. The fluorescein test strip was inserted in the patient’s lower eyelid’s conjunctival sac, and the patient was asked to blink 3–4 times to fully mix the fluorescein with their tears. The tear film breakup time was determined from the natural primary gaze opening until the first black spot appeared in the cornea (14). This operation was repeated three times, and an average was recorded.
(4) CFS. Following the procedure of fluorescein staining, cobalt blue light was used to record the staining status. According to the NEI evaluation criteria (4), the degree of fluorescein staining was scored in the central, upper, temporal, nasal and lower parts of the cornea, and the total score was calculated.
(5) Schirmer I test. A Schirmer test strip was inserted into the conjunctival sac in the inner 1/3 part of the lower eyelid of the tested eye. The patient was told to gently close the eye for 5 min, and the test paper was taken out to read the length of the tear wet area (14).
(6) Intraocular pressure. Non-contact intraocular pressure measurement was adopted.

In order to avoid mutual interference among examination results, our examination sequence was visual acuity, questionnaire survey, slit lamp examination, BUT, CFS, Schirmer I examination (performed at least 30 min after the CFS test) and non-contact intraocular pressure examination.

(7) Safety analysis

In this trial, all adverse events and other safety data were collected. MedDRA Ver.23.0 was used for statistical analysis of systemic and ocular adverse events as well as ADRs. The incidence rates of ADRs were determined based on the entire population who had received drug treatment: incidence = number of cases of ADRs/number of patients. The severity of ADRs was classified into mild, moderate, and severe based on its scope or degree. Mild cases had symptoms and signs but were completely tolerable; moderate symptoms and signs would cause discomfort to the patient and affect normal activities of daily living; severe symptoms and signs would prevent the patient from working or performing daily activities.

### Statistical analysis

Statistical analysis was conducted with SAS (Ver.9.4, SHANGHAI DATANINE SOFTWARE CO., LTD). The data distributions in this study were tested by Kolmogorov–Smirnov test using SAS 9.4 to assure the variables indeed follow normal distribution. Quantitative data were described by number, mean and standard deviation (SD); 95% confidence intervals for the means were also calculated. Qualitative data were described as number and percentage. One-way analysis of variance was used for comparisons among three groups. Two-side *t*-test was used for comparisons between two groups. Details of statistics were shown in figure legends and tables. The null hypothesis was not rejected when the value of *p* > 0.05.

## Results

### Patients

**Figure 1:**
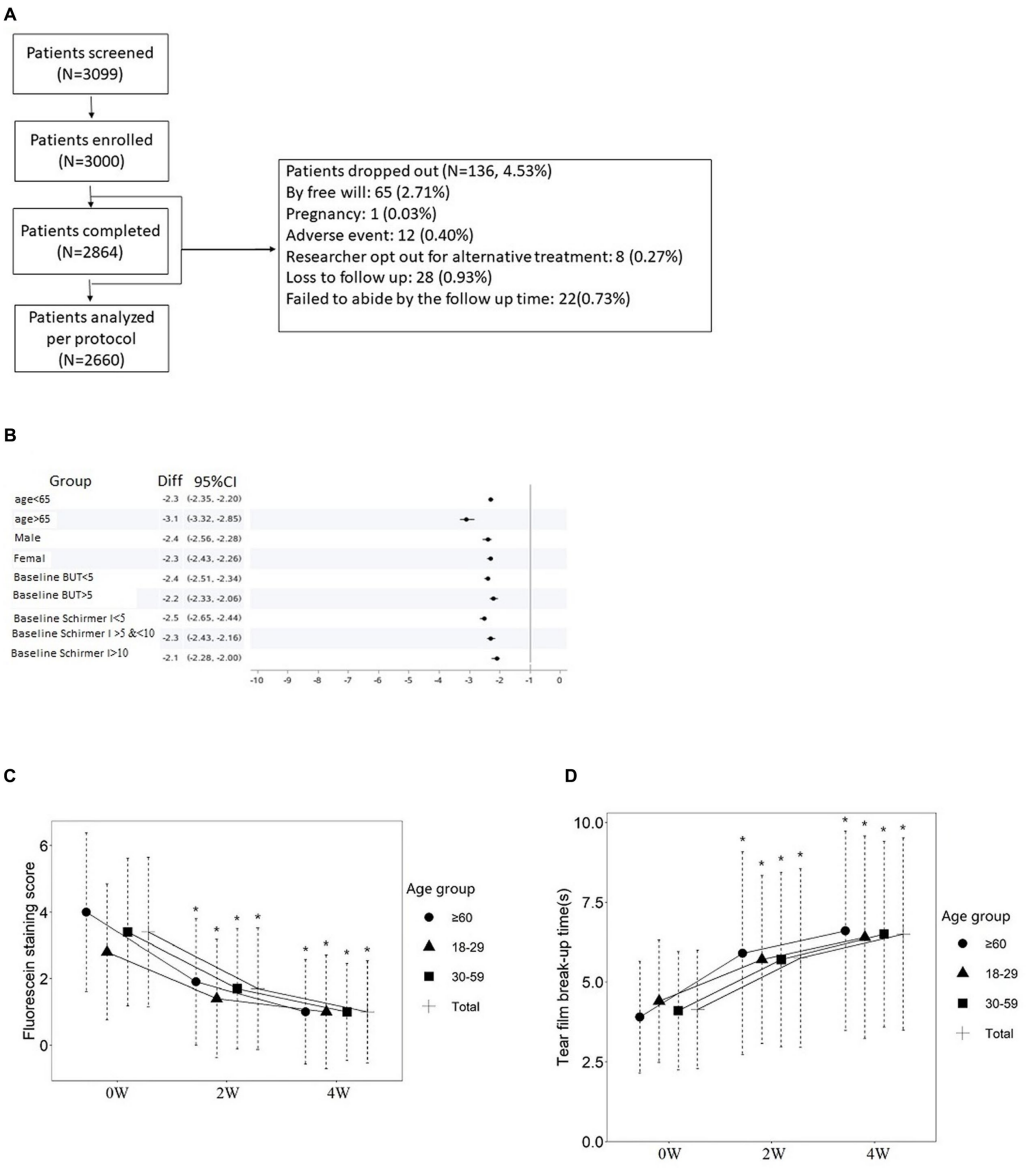
Dry eye treatment with 3% diquafosol eye drops. **(A)** Flow chart of patient screening and enrollment of the study, dropouts are included. **(B)** Forest plot of baseline CFS of the entire cohort. **(C)** CFS change in different age groups and the cohort total, *p < 0.05. **(D)** Tear film break up time in different age groups and the cohort total, *p < 0.05.

From Mar 2019 to Jan 2022, 3,099 patients were screened, 3,000 met the criterion and were enrolled in the study. A total of 2,864 (95.47%) patients completed the trial, and 2,660 abided by the protocol in follow ups. A total of 136 (4.53%) patients dropped out and the drop-out reasons are shown in Figure 1A.

Baseline demographic and clinical characteristics are shown in Table 1. Compared to the phase III cohort we reported in 2016 (13), we were able to expand the cohort to contain more elderly patients. We further segregated patients according to their age, gender and history (Table 1), for example, for age characteristics, the cohort was segregated into three groups: Group A (≥18 ≤ 29 years old, *n* = 593), B (≥30 ≤ 59 years old, *n* = 1,543), and C (≥60 years old, *n* = 524). Figure 1B shows the forest plot of the baseline CFS characteristics (judged by fluorescein staining results) for different subgroups. To make sure patients’ basic eye function was not negatively impacted by the whole procedure, visual acuity examination and ocular pressure were included in the follow up data recording. The baseline visual and ocular characteristics of all patients are shown in Table 2. When the patient age increases, we find the absolute CFS tended to increase (Table 1, Figure 1B), while SIT and BUT values tended to decrease (Table 1). Visual acuity results at baseline were 0.19 ± 0.28. Baseline IOp values were 14.88 ± 2.92 mmHg (Table 2).

**Table 1:** Age, gender, history segregated clinical characteristics from complete follow up.

Group	BUT mean ± standard deviation [sec]			*p*-value^II^ among segre-gated group	CFS mean ± standard deviation			*p*-value^II^ among segre-gated group	Schirmer I mean ± standard deviation [mm/5 min]			*p*-value^II^ among segre-gated group
	0 W	2 W	4 W		0 W	2 W	4 W		0 W	2 W	4 W	
Total population (*N* = 2,660)	4.14 ± 1.85	5.75 ± 2.80	6.50 ± 3.01		3.40 ± 2.25	1.70 ± 1.83	1.00 ± 1.54		9.10 ± 8.14	5.75 ± 2.80	6.50 ± 3.01	
Group A: ≥18 ≤ 29 ys (*N* = 593)	4.40 ± 1.90	5.70 ± 2.63 (p* = 0.001)	6.40 ± 3.17 (p* = 0.001)	*p*^II^ = 0.0001	2.80 ± 2.04	1.40 ± 1.78 (p* = 0.001)	1.00 ± 1.71 (p* = 0.001)	*p*^II^ = 0.0001	13.00 ± 9.76	11.90 ± 8.76 (p* = 0.001)	12.10 ± 8.84 (p* = 0.001)	*p*^II^ = 0.007
Group B: ≥30 ≤ 59 ys (*N* = 1,543)	4.10 ± 1.85	5.70 ± 2.73 (p* = 0.001)	6.50 ± 2.91 (p* = 0.001)		3.40 ± 2.22	1.70 ± 1.81 (p* = 0.001)	1.00 ± 1.46 (p* = 0.001)		8.50 ± 7.63	9.70 ± 7.25 (p* = 0.001)	10.00 ± 7.12 (p* = 0.001)	
Group C: ≥60 ys (*N* = 524)	3.90 ± 1.75	5.90 ± 3.18 (p* = 0.001)	6.60 ± 3.12 (p* = 0.001)		4.00 ± 2.39	1.90 ± 1.90 (p* = 0.001)	1.00 ± 1.58 (p* = 0.001)		6.50 ± 5.67	8.80 ± 5.63 (p* = 0.001)	9.60 ± 5.70 (p* = 0.001)	
Sex: Male (*N* = 680)	4.23 ± 1.85	6.05 ± 2.89 (p* = 0.001)	6.88 ± 3.18 (p* = 0.001)	*p*^II^ = 0.6469	3.30 ± 2.21	1.60 ± 1.74 (p* = 0.001)	0.90 ± 1.47 (p* = 0.001)	*p*^II^ = 0.29	7.90 ± 7.16	9.00 ± 6.42 (p* = 0.000)	9.60 ± 6.56 (p* = 0.000)	*p*^II^ = 0.8944
Female (*N* = 1,980)	4.10 ± 1.85	5.65 ± 2.77 (p* = 0.001)	6.37 ± 2.94 (p* = 0.001)		3.40 ± 2.27	1.70 ± 1.86 (p* = 0.001)	1.00 ± 1.56 (p* = 0.001)		9.50 ± 8.42	10.30 ± 7.68 (p* = 0.000)	10.50 ± 7.56 (p* = 0.000)	
Endo-crine disorders: Yes (*N* = 27)	3.70 ± 1.70	5.40 ± 1.74 (p* = 0.002)	5.80 ± 2.41 (p* = 0.001)	*p*^II^ = 0.8921	3.50 ± 1.76	2.00 ± 1.74 (p* = 0.001)	1.00 ± 1.13 (p* = 0.001)	*p*^II^ = 0.73	7.30 ± 5.98	6.50 ± 5.43 (p* = 0.001)	7.80 ± 6.24 (p* = 0.001)	*p*^II^ = 0.003
Endo-crine disorders: No (*N* = 2,633)	4.10 ± 1.85	5.80 ± 2.81 (p* = 0.001)	6.50 ± 3.01 (p* = 0.001)		3.40 ± 2.26	1.70 ± 1.83 (p* = 0.001)	1.00 ± 1.55 (p* = 0.001)		9.10 ± 8.16	10.10 ± 7.43 (p* = 0.001)	10.40 ± 7.36 (p* = 0.001)	
History of eye surgery: Yes (*N* = 63)	4.10 ± 2.37	4.74 ± 2.51 (p* = 0.0026)	5.30 ± 2.72 (p* = 0.003)	*p*^II^ = 0.4604	2.90 ± 2.08	1.30 ± 1.61 (p* = 0.001)	0.80 ± 1.32 (p* = 0.001)	*p*^II^ = 0.5656	10.30 ± 9.88	11.60 ± 9.94 (p* = 0.5048)	9.60 ± 9.56 (p* = 0.4081)	*p*^II^ = 0.007
History of eye surgery: No (*N* = 2,597)	4.15 ± 1.84	5.77 ± 2.81 (p* = 0.001)	6.53 ± 3.01 (p* = 0.001)		3.40 ± 2.26	1.70 ± 1.83 (p* = 0.001)	1.00 ± 1.55 (p* = 0.001)		9.10 ± 8.10	10.00 ± 7.34 (p* = 0.000)	10.40 ± 7.29 (p* = 0.000)	
History of eye drop (*N* = 81)	4.21 ± 1.71	5.79 ± 2.57 (p* = 0.001)	6.19 ± 2.96 (p* = 0.001)		3.70 ± 2.50	1.70 ± 2.18 (p* = 0.001)	1.10 ± 1.62 (p* = 0.001)		10.40 ± 9.60	9.50 ± 8.60 (p* = 0.4163)	10.20 ± 9.32 (p* = 0.9096)	

* *p*-value for the difference towards baseline condition was calculated by *t*-test.

II *p*-value for the difference among 4 weeks follow up results of segregated subgroups was calculated by ANOVA.

**Table 2:** Visual acuity and intraocular pressure.

	Uncorrected visual acuity of the study eye	*p*-value relative to baseline	Corrected visual acuity of the study eye	*p*-value relative to baseline	Non-contact intraocular pressure	*p*-value relative to baseline
	Mean ± SD		Mean ± SD		Mean ± SD	
0 W	0.194 ± 0.283		0.064 ± 0.133		14.88 ± 2.92	
2 W	0.185 ± 0.279	0.0001*	0.056 ± 0.125	0.0001*	14.62 ± 2.85	0.0001*
4 W	0.176 ± 0.271	0.0001*	0.051 ± 0.122	0.0001*	14.59 ± 2.87	0.0001*

* *p*-value for the difference towards baseline condition was calculated by *t*-test.

### Primary outcome

We followed up CFS from baseline to 2- and 4-weeks after prescribed treatment. Compared to baseline condition, CFS scores at 2 weeks and 4 weeks of treatment were decreased from 3.4 ± 2.25 to 1.7 ± 1.83 and 1.0 ± 1.54 (Table 1). These changes were statistically significant compared to the ones at baseline (*p* < 0.05) (Table 1, Figure 1C). At 2 weeks of treatment, corneal staining was improved in 2,413 (83.29%) patients and turned negative in 949 (32.76%) (Figure 1C). At 4 weeks of treatment, corneal staining score was improved in 2,627 (90.65%) patients and turned negative (i.e., no break in fluorescein staining image) in 1,504 (51.90%) (Figure 1C).

After 2- and 4-weeks treatment with 3% diquafosol sodium eye drops, a decrease of CFS is observed in all segregated groups compared to their baseline scores (Table 1). At 2 weeks of treatment, the total score difference vs. baseline was −1.7, with −1.41 in Group A (95% CI, −1.53, −1.30%), −1.71 in Group B (95% CI, −1.79, −1.62%), and − 2.13 in Group C (95% CI, −2.28, −1.97%); showing a slight better improvement in elderly patients Group C. At 4 weeks of treatment, the total score difference was −2.4, with −1.84 in Group A (95% CI, −1.98, −1.70%), −2.41 in Group B (95% CI, −2.51, −2.32%), and − 3.02 in Group C (95% CI, −3.21, −2.84%), more significant beneficial effect was observed in the most elderly group, and this observation is statistically significant (*p* < 0.05, Table 1).

We analyzed efficacy by gender and there were 680 males and 1,980 females. At 2 weeks of treatment, CFS differences versus baseline were −1.7 for males and −1.7 for females; at 4 weeks of treatment CFS differences versus baseline were −2.5 for males and −2.4 for females. There is no gender difference (*p* > 0.05, Table 1).

### Secondary outcomes

For secondary outcomes, we followed up with the changes in CFS score in patients with prior artificial tear treatment, a history of eye surgery and a history of endocrine disease. In addition, we also followed up with BUT and Schirmer I test results.

A total of 81 patients had used other artificial tears for dry eye treatment for more than 1 week before the enrollment and switched to 3% diquafosol sodium eye drops after the enrollment. The improvement of corneal fluorescein score at 2- and 4-weeks over baseline were all statistically significant (Table 1).

Sixty-three patients had a history of eye surgery, including refractive surgery, cataract surgery and vitreous surgery. Among these, 55 refractive surgeries were performed, accounting for 74.3% (55/63). Compared with baseline, the differences in corneal fluorescein score improvement in these patients at 2 weeks and 4 weeks of treatment were all statistically significant (Table 1).

Twenty-seven patients had accompanying endocrine diseases, including thyroid nodules, hypothyroidism, hyperthyroidism, thyroiditis and hypopituitarism. Compared to the patients without endocrine diseases, 3% diquafosol sodium eye drops show similar efficacy in cases with a history of endocrine diseases (Table 1).

For tear film breakup time, compared to baseline value, BUT values at 2- and 4-week of treatment were significantly improved (*p* < 0.05) in each age group (Figure 1D). Interestingly a more significant improvement in BUT in elderly group is again observed (*p* < 0.05, Table 1). At 4 weeks of treatment, the total improvement of BUT was 2.38, with 2.00 in the age group of 18–29 years old (Group A, 95% CI, 1.76, 2.24%), 2.35 in the age group of 30 to 59 years old (Group B, 95% CI, 2.22, 2.48%) and 2.76 in 60 years and older (Group C, 95% CI, 2.52, 3.01%).

For SIT results, compared with baseline, the differences in SIT results at 2 weeks and 4 weeks of treatment in each age group were all significant (*p* < 0.05, Table 1).

### Safety

After 2 weeks and 4 weeks of treatment with 3% diquafosol sodium eye drops, the logarithmic values of uncorrected visual acuity (UCVA) as well as corrected visual acuity (CVA) were higher versus baseline (*p* < 0.05, Table 3).

During the entire study period, a total of 185 patients experienced 244 cases (6.17%) of ADRs (Table 3). Among them, there were 233 cases of ocular ADRs (180 patients, 6.00%) and 11 of systemic ADRs (9 patients, 0.30%). Of all ADRs, 91.80% were mild and 8.20% were moderate; there were no severe or rarer ADRs. The outcomes of ADRs were recovery in 89.75%, improvement in 9.40% and no improvement in 0.80%. The average time to recovery of ADRs was 15.60 ± 13.13 (1–73) days.

**Table 3:** Summary of ADRs during treatment.

	3% dexamethasone sodium eye drops (*N* = 3,000)			
MedDRA preferred term	Case number	Number of cases	Incidence (%)	95% confidence interval
ADRs	244	185	6.17	(5.33, 7.09%)
Ocular ADRs	233	180	6.00	(5.18, 6.91%)
Eye discharge	58	58	1.93	(1.47, 2.49%)
Eye pain	39	39	1.30	(0.93, 1.77%)
Foreign body sensation in eyes	26	26	0.87	(0.57, 1.27%)
Eye pruritus	20	20	0.67	(0.41, 1.03%)
Eye irritation	17	17	0.57	(0.33, 0.91%)
Ocular hyperemia	14	14	0.47	(0.26, 0.78%)
Lacrimation increased	9	8	0.27	(0.12, 0.52%)
Blurred vision	7	7	0.23	(0.09, 0.48%)
Abnormal sensation in eye	7	7	0.23	(0.09, 0.48%)
Conjunctivitis	4	4	0.13	(0.04, 0.34%)
Conjunctivitis allergic	4	4	0.13	(0.04, 0.34%)
Keratitis	4	4	0.13	(0.04, 0.34%)
Systemic ADRs	11	9	0.30	(0.14, 0.57%)
Dizziness	4	4	0.13	(0.04, 0.34%)

ADRs report by ≥ 0.1%.

Incidence = number of cases/number of subjects; the calculation of percentage was based on all drug-experienced patients.

Most ADRs occurred soon after the initial dose. A total of 91 cases of ADRs (37.3%) occurred on the first day after treatment, 160 (65.57%) (mainly eye discharge and eye pain) occurred within 7 days after treatment, and 5 (2.49%) occurred within 30 days after treatment. The adverse event was followed up till 4 weeks after the treatment, since the long term reactions (eye discharge, eye irritation and eye pain) had been reported in a prior Japanese report (15). Totally 90 patients experienced 97 cases (3%) of >grade 1 ocular ADRs. There were 58 cases (1.93%) of ocular secretions and 39 (1.30%) of eye pain.

A total of 41 patients experienced 57 cases (1.37%) of ADRs leading to a dropout or physician opt out decision (Figure 1A). Of these, 37 patients experienced 51 cases (1.23%) of ocular ADRs, they were mostly ocular infection or allergic reactions. Five patients experienced 6 cases (0.09%) of systemic ADRs, and 4 of them were self-reported dizziness.

In addition, the complaints we received from the dropped-out patients were foreign body sensation in the eye (*n* = 10, 11.6%), ocular hyperemia (*n* = 6, 7%), eye itching (*n* = 6, 7%) and eye pain (*n* = 6, 7%). After using the study drug, one patient without a history of glaucoma had increased intraocular pressure of 24 mmHg at 1 month, which further increased to 27 mmHg on the following day. Since the IOP was increased after 4 weeks of treatment, he had completed the trial. Therefore, the patient was treated with timolol maleate ophthalmic solution twice a day. The intraocular pressure returned to normal after 1 month of continuous medication of timolol maleate ophthalmic solution. This patient was extensively followed up, and his intraocular pressure remained normal at 2 months after withdrawal of timolol maleate ophthalmic solution.

## Discussion

In this real-world study we assessed the efficacy and safety of 3% diquafosol sodium eye drops in the treatment of Chinese patients with dry eye symptoms (2). The results showed that dry eye symptoms were quickly relieved after 2 weeks of treatment with 3% diquafosol sodium eye drops, and a further minor improvement was also observed during subsequent treatment. There was a statistically significant difference in CFS score compared with baseline condition, indicating that 3% diquafosol sodium eye drops can quickly promote corneal epithelial repair and restore the integrity of the ocular surface. And we are excited to report this improvement is more significant in the elderly group (Group C, >60 years old). The possible mechanism is that diquafosol sodium promotes mucin secretion from goblet cells after binding to the corresponding receptor, so it can promote the repair of the corneal epithelium under the protection of mucin. Therefore, 3% diquafosol sodium eye drops can significantly reduce corneal fluorescein staining. A phase III clinical trial jointly conducted in China and Singapore compared the therapeutic effects of diquafosol sodium eye drops and sodium hyaluronate eye drops in nearly 500 dry eye cases in parallel (13). The results showed that the improvement of ocular surface Rose Bengal staining in the diquafosol sodium eye drops group was superior to sodium hyaluronate group, and both groups had similar results in terms of corneal fluorescence staining improvement. This study further expanded the insights from several aspects (1) that the use of 3% diquafosol sodium eye drops for dry eye can effectively increase tear secretion, prolong BUT, reduce CFS; (2) there was an obvious effect at 2 weeks of treatment, and the efficacy continued to strengthen with subsequent use till 4 weeks. It is an effective drug for the treatment of dry eye; (3) the elderly group with age older than 60 years had the most significant improvement judged by CFS and BUT.

The function of the lachrymal functional unit declines while aging (16). At the meantime, dual regulation of endocrine hormone secretion and somatosensory nerve conduction causes a cascade of ocular surface inflammation and changes in osmotic pressure, and the symptoms and signs of dry eye gradually worsen (5). In the lacrimal gland of the elderly, T cells that react automatically due to inflammation can be detected, suggesting that age is not only a simple degenerative process, but also involves inflammatory responses. In this study, the enrolled patients were divided into three groups by age, and clinical characteristics of different age groups at baseline and follow up in treatments were compared. The elderly benefit from the treatment the most. A prospective cross-sectional study explored the association between aging and the natural history of dry eye showed that the risk of dry eye increased by 24% for every 10 years of aging (7).

After treatment with 3% diquafosol sodium eye drops, there were differences in the improvement of clinical characteristics in 3 age groups, which may be due to age related differentiated responses of goblet cells and corneal and conjunctival epithelial cells. Some reports proposed that age is a predictor of inconsistency between the symptoms and signs of dry eye (17, 18). Although the symptoms of dry eye in the elderly are more severe (7), there is no correlation between aging and the ocular surface discomfort index, which may be related to decreased corneal sensitivity in the elderly. In this study, since we used a tear secretion evaluation test without surface anesthesia, the SIT values contained significant variance and especially in younger group of patients, which made the results hard to interpret.

Sex hormones are associated with the progression of many ocular surface diseases, including dry eye, meibomian gland dysfunction, wound healing and keratoconjunctivitis (19–22). The reduction of sex hormones such as androgens is one of the reasons for the high incidence of dry eye in the elderly group. In addition, female prone factors, such as chronic pain or other iatrogenic dry eye risk factors also increase the prevalence of female dry eye cases (11). Asian ethnicity is another risk factor for dry eye (7). The proportion of women enrolled in this study was relatively high, which was consistent with other research reports (23). However, after treatment with 3% diquafosol sodium eye drops, both male and female groups had good responses, and differences in Schirmer, BUT and CFS before and after treatment were not significantly different between male or female. Studies have reported that as age increases, the gender difference in dry eye incidence becomes smaller, which means clinical manifestations in male and female patients in older people are closer to each other (24).

Systemic disease is a high-risk factor for dry eye symptoms. In this trial, 35 patients had a history of endocrine diseases. The main manifestation was abnormal thyroid function. In this study, there were no differences in baseline BUT, Schirmer I and CFS between patients with a history of endocrine diseases and those without, and there were no differences between the therapeutic effects of the two groups. Yu et al. (25) reported that the severity of dry eye is significantly correlated with Sjogren, facial erythema, rheumatoid arthritis, and peripheral arterial obstructive disease, and not associated with abnormal thyroid function, osteoarthritis, diabetes, hypercholesterolemia, hypertension, irritable bowel syndrome and hyperuricemia. Other studies suggested that patients with abnormal thyroid function accompanying thyroid eye disease are more likely to develop dry eye compared with individuals without thyroid eye disease (26). The patients in this study with a history of endocrine disorders, such as thyroid disorders, did not show obvious signs of systemic diseases; in this case, the disease might be in the early stage, and a vicious cycle of neural reflexes or autoimmune loops in the eye aggravating dry eye has not been established. Therefore, there was no difference between the two groups. Future studies are warranted to analyze the differences in tear film osmotic pressure and MMP-9 and to investigate whether inflammation is a predictive factor of dry eye in patients with endocrine disorders, which may help formulate treatment plans for this subgroup of patients.

Corneal punctate epithelial staining is a recommended diagnosis criteria for dry eye (2). The inflammation associated with dry eye can cause epithelial cell damage, loss of goblet cells and interference with mucin secretion, and consequently corneal epithelial staining. The scope of fluorescent staining is critical because it shows how much the normal tissue remains to compensate for the loss of cells in the damaged area (2). In cases of sufficient healthy epithelium, the damaged tissue can be replaced by normal cells; however, when the epithelium damage area is too large, inflammation and scarring will affect the healing of mucosal tissues (5). In this study, CFS was selected as the primary measurement to determine whether 3% diquafosol sodium eye drops could accelerate the repair of superficial corneal damage, and the therapeutic efficacy (27). The clinical observation results also proved that 3% diquafosol sodium eye drops had similar efficacy to conventional artificial tears (28).

Sixty-three patients had a history of eye surgery before enrollment and acquired improvement in dry eye symptoms and signs. The results demonstrated that those patients could also benefit from 3% diquafosol sodium eye drops treatment. Since most eye operations were refractive surgery for myopia, in addition to corneal nerve damage, wearing contact lenses for a long time before surgery was one of the reasons for severe dry eye symptoms and poor therapy prognosis in these patients. Future trials should carefully assess the state of dry eye before refractive surgery and investigate the influence of other factors on surgically induced dry eye.

The current study also showed that the uncorrected visual acuity in dry eye cases was slightly improved after 4 weeks of medication, which may be due to an increased tear film stability and negative conversion of corneal staining. Intraocular pressure was slightly decreased in patients, which may be related to intraocular pressure improvement after a successful repair of the tear film. The results of visual acuity and intraocular pressure tests also indirectly demonstrated the good safety of 3% diquafosol sodium eye drops. During the treatment, no serious ADRs occurred. The main discomfort in patients was mild tingling and burning sensation after application of the eye drops.

## Discussion of limitation

A total of 30 medical institutions in Mainland China participated in this study. Due to differing configuration of the testing equipment in various centers, specific tests such as non-contact BUT and tear osmotic pressure were not performed.

## Conclusion

To summarize, the overall results showed that 3% diquafosol sodium eye drops had excellent effects in improving the repair of corneal epithelial damage, increasing tear secretion, prolonging tear film breakup time, and alleviating other symptoms. Future studies may consider including more comprehensive tear examination methods and ocular surface analysis.

## Data availability statement

The datasets presented in this study can be found in online repositories. The names of the repository/repositories and accession number(s) can be found in the article/Supplementary material.

## Ethics statement

The studies involving human participants were reviewed and approved by this study (Chinese Clinical Trial Registry ID: ChiCTR1900021999, Registration Date: 19/03/2019) was approved by the Ethics Committee of Beijing Tongren Hospital, Capital Medical University (approval number: TREC2018-50). All patients provided signed informed consent, and the study followed the Declaration of Helsinki. The patients/participants provided their written informed consent to participate in this study.

## Author contributions

WW had full access to all of the data in the study and take responsibility for the integrity and accuracy of the data, performed acquisition, analysis, or interpretation of data; drafting of the manuscript; critical revision of the manuscript for important intellectual content; statistical analysis. XS and LL data acquisition. All authors: acquisition. All authors contributed to the article and approved the submitted version.

## Conflict of interest

This clinical trial was sponsored by Santen Pharmaceutical (China) Co., Ltd. They had no role in the design or conduct of this research.

The authors declare that the research was conducted in the absence of any commercial or financial relationships that could be construed as a potential conflict of interest.

## Publisher’s note

All claims expressed in this article are solely those of the authors and do not necessarily represent those of their affiliated organizations, or those of the publisher, the editors and the reviewers. Any product that may be evaluated in this article, or claim that may be made by its manufacturer, is not guaranteed or endorsed by the publisher.
